# Retraction Note: Tripartite motif-containing 14 (TRIM14) promotes epithelial-mesenchymal transition via ZEB2 in glioblastoma cells

**DOI:** 10.1186/s13046-023-02646-9

**Published:** 2023-03-28

**Authors:** Shuang Feng, Xiaomin Cai, Yangyang Li, Xiaoguang Jian, Linxin Zhang, Bin Li

**Affiliations:** 1 Department of Encephalopathy, The Third Affiliated Hospital of Nanjing University of Chinese Medicine, Nanjing, Jiangsu China; 2 Department of Neurosurgery, The First Affiliated Hospital of Nanjing Medical University, Nanjing, Jiangsu China

**Retraction Note:** **J Exp Clin Cancer Res 38, 57 (2019)**

**https://doi.org/10.1186/s13046-019-1070-x**

The Editor-in-Chief has retracted this article. Concerns were raised regarding Figure 4B: the LN229 and U251 ZEB2 bands appear to be identical but flipped horizontally. Additionally, the authors have been unable to provide convincing documentation that ethics approval was obtained for this study. The Editor-in-Chief therefore no longer have confidence in the results and conclusions of this article.

Author Shuang Feng disagrees with this retraction. The other authors have not responded to correspondence regarding this retraction.

